# Patterns of Brain Injury and Clinical Outcomes Related to Trauma from Collisions Involving Motor Vehicles

**DOI:** 10.3390/jcm13247500

**Published:** 2024-12-10

**Authors:** Bharti Sharma, Aubrey May B. Agcon, George Agriantonis, Samantha R. Kiernan, Navin D. Bhatia, Kate Twelker, Zahra Shafaee, Jennifer Whittington

**Affiliations:** 1 Department of Surgery, NYC Health and Hospitals, Elmhurst Hospital Center, New York, NY 11373, USA; bokoa1@nychhc.org (A.M.B.A.); agriantg@nychhc.org (G.A.); bhatian1@nychhc.org (N.D.B.); harrisj20@nychhc.org (J.W.); 2 Department of Surgery, Icahn School of Medicine, Mount Sinai Hospital, New York, NY 10029, USA; shafaeez1@nychhc.org; 3 Touro College of Osteopathic Medicine–Harlem, New York, NY 10027, USA; skiernan@student.touro.edu

**Keywords:** traumatic brain injury, motor vehicle collisions, severity, injury patterns

## Abstract

**Background**: Despite improvements in technology and safety measures, injuries from collisions involving motor vehicles (CIMVs) continue to be prevalent. Therefore, our goal is to investigate the different patterns of head injuries associated with CIMVs. **Method**: This is a single-center, retrospective study of patients with motor vehicle-related trauma between 1 January 2016–31 December 2023. Patients were identified based on the International Classification of Diseases (ICD) injury codes and the Abbreviated Injury Severity (AIS) for body region involvement. **Result**: 536 patients met the inclusion criteria. The majority of the injured population includes pedestrians (46.8%), followed by motorcycle drivers (25.2%), bicyclists (18.7%), and motor vehicle drivers (9.3%). The male-to-female ratios for bicyclists and motorcyclists were 13.7:1 and 11.9:1, respectively, which is higher compared with motor vehicle occupants and pedestrians, with ratios of 2.3:1 and 1.5:1. Patients with blunt trauma (99.63%) were higher than penetrating trauma (0.37%). In most cases, the head had the highest AIS score, with a mean of 3.7. Additionally, the median Injury Severity Score (ISS) was 20. Skull fractures were the most prevalent, followed by hemorrhages, lacerations, contusions, and abrasions. **Conclusions**: The most prevalent injuries were head injuries and fractures. Fractures were the most common, followed by hemorrhage, laceration, contusion, and abrasion. These findings underscore the high incidence of TBI and fractures in such CIMVs, highlighting the need for targeted trauma interventions and the need for injury prevention strategies to mitigate these severe outcomes.

## 1. Introduction

Collisions involving motor vehicles (CIMVs) remain a leading cause of traumatic injury and mortality worldwide [1], causing more than 1 million deaths and 50 million injuries annually [2], presenting a critical public health challenge. According to the Global Burden of Disease report, road injuries were the tenth leading cause of disease burden worldwide in 2021 [3] and were the ninth most common cause of disability-adjusted life-years lost for all ages and categories [4]. In the United States, there were more than 3.5 million visits to the emergency department because of motor vehicle-related injuries in 2010–2011 [5]. Despite significant advancements in vehicle safety, urban infrastructure, and traffic regulations, the complexity and severity of injuries from collisions involving motor vehicles continue to strain trauma centers; CIMVs make up one of the highest proportions of trauma-related injuries in higher-income countries [6]. Head injuries specifically are strongly tied to transportation, with car accidents being one of the leading causes of traumatic head and spinal cord injury [7].

New York City, known for being one of America’s most walkable cities, faces unique challenges, with over 60% of commuters walking or using public transportation as of 2016 [8]. This is in contrast to the rest of the country, of which 84.8% use a car, truck, or van as their primary means of transportation [9]. Even when compared to other major metropolitan cities in the United States, New York City stands out, comprising 38.7% of all public transportation commuters in the United States with the next highest prevalence in Chicago at only 7.5% [9]. Though New York has one of the lowest mortality rates related to motor vehicle injury [10], it still presents a significant problem. In a systematic review of high-income countries, the incidence of pedestrian traffic injury was higher in the United States compared to Europe and was the highest in New York City out of all cities studied [11]. Furthermore, though New York City has seen significant decreases in traffic injuries over recent decades due to the implementation of programs such as Vision Zero, the impacts of these improvements were reversed during the COVID-19 pandemic [12].

Previous studies have investigated the epidemiology and outcomes of injuries related to CIMVs. However, in-depth analyses still need to focus on specific types of transportation, such as pedestrians, motorcyclists, bicyclists, and vehicle occupants, and their relation to each other. Each group displays unique injury patterns and clinical outcomes, which can help shape targeted prevention strategies and optimize resource allocation within trauma care systems. Additionally, contextualizing each group with each other instead of studying each transportation mode in isolation allows for additional patterns to be elicited.

This study aims to examine the injury patterns and clinical outcomes associated with CIMVs among patients presenting to a level I trauma center in New York City. By analyzing data from various transportation modalities, the research provides valuable insights into how different forms of transportation affect the type and severity of trauma, as well as patient outcomes. The findings will help guide future injury prevention efforts and improve patient care. This analysis is intended to inform trauma care protocols, public health strategies, and injury prevention initiatives, particularly in urban areas with high traffic density and pedestrian activity. This retrospective study was approved by the IRB at our facility on 24 October 2024, with IRB number 24-12-380-05G. Informed consent was waived due to the retrospective nature of this study.

## 2. Methods

This is a single-center, retrospective review conducted at a level 1 trauma center verified by the American College of Surgeons in Queens, New York City. We included all patients who presented with a severe traumatic brain injury as a result of a collision with a motor vehicle between 1 January 2016 and 31 December 2023, inclusive. Patient data were requested from the National Trauma Registry of the American College of Surgeons (NTRACS) Database at our center (Elmhurst Hospital Center). Patients were identified based on the injury mechanism, cause of injury, primary mechanisms (lCD9 or lCDL0 E-Code), and the Abbreviated Injury Severity (AIS) score on the head. The AIS score ranges from 1 to 6 per body region. Based on trauma registry records, we identified 536 patients with severe TBI involving traumatic injuries as a result of a collision involving a motor vehicle. The patients’ medical charts were reviewed, and all relevant information required for this study was collected.

We excluded data points incompatible with our study, including patients who died within the first 24 h of admission, those with an AIS score of less than 3, patients with non-motor vehicle-related injuries, and individuals with non-severe or minor injuries. All other patients were included except for these exclusions. In this included patient population, there were no missing data points. We utilized two sources to obtain complete information on the patient population. First, we requested protected health information (PHI) and other relevant data elements from NTRACS, our center’s trauma registry. If the registry did not include a specific data point for any patient, we used PHI, such as the medical record number (MRN) and the patient’s name, to review their chart and obtain the missing information.

We collected data using a data collection tool (Excel sheet or spreadsheet). We incorporated all data elements into this tool. Examples of data elements are demographics (for example, age, sex, race, and ethnicity), AIS, traumatic brain injury pattern, discharge disposition, mortality status, and others. The dataset underwent several preprocessing steps to ensure data integrity, confidentiality, and suitability for statistical analysis. First, to de-identify the dataset, unique identifiers such as medical record numbers (MRNs), dates of birth (DOBs), and patient names were removed. This process was performed to maintain patient privacy in compliance with ethical research standards. Next, inclusion and exclusion criteria were followed, as discussed above, followed by data analysis.

Data analysis was performed using the Python programming language in the Google Colab environment (version 1.0.0), which offers a cloud-based platform for reproducible computational workflows. The dataset included binary-coded variables for the dependent variable (discharge outcome) and various independent variables, such as severe TBI-related injuries. The dependent variable was encoded as 0 for negative outcomes and 1 for positive outcomes. The independent variables indicated the presence or absence of specific injuries, facilitating a logistic regression analysis.

The dataset was first inspected for missing values and inconsistencies, with appropriate cleaning performed as needed. Features were scaled and encoded where necessary to ensure compatibility with the logistic regression algorithm. Given the binary nature of the data, exploratory data analysis included calculating distributions of the dependent and independent variables and evaluating class imbalances.

Three logistic regression models were implemented using the statsmodels library. The first model served as a baseline and was built without any adjustments. The second model applied class weighting to address the imbalance between positive and negative outcomes. The third model incorporated the Synthetic Minority Oversampling Technique (SMOTE) using the imbalanced-learn library to generate synthetic samples for the minority class, improving the model’s ability to predict positive outcomes. A Random Forest classifier was included as a benchmark non-linear model to evaluate its performance compared to the logistic regression models.

Model performance was evaluated by analyzing Receiver Operating Characteristic (ROC) curves and their corresponding Area Under the Curve (AUC) values. Additionally, we calculated other metrics, including precision, recall, F1-score, and overall accuracy, using the sklearn library to assess the balance between sensitivity and specificity. To visualize the results, we created comparative plots of the ROC curves and metrics using Matplotlib.

Independent variables were not mutually exclusive, reflecting overlapping injury patterns identified in the electronic medical records. This overlap was accounted for in the analysis through the inherent flexibility of logistic regression and Random Forest algorithms. Given the class imbalance in the dataset, optimizations such as class weighting, SMOTE, and algorithmic adjustments were applied to ensure reliable and meaningful results.

### Software and Reproducibility

All analyses were performed using Python (version 3.10.12) in the Google Colab environment. The following libraries and versions were used: statsmodels (0.13.5), scikit-learn (1.3.1), and imbalanced-learn (0.10.1). The code and data preprocessing steps were designed to ensure reproducibility, with annotated scripts available for further validation and replication of results. The above computational approach allowed for efficient handling of binary-coded clinical data and enabled the comparison of multiple predictive models to identify the best-performing approach for analyzing severe TBI-related injuries and discharge outcomes.

## 3. Results

### 3.1. Association Between Demographics and the Mode of Transportation

A total of 536 patients met the inclusion criteria. These patients were further differentiated based on the mode of transportation associated with their injury. It was found that pedestrian injuries were most prevalent with 251 patients (46.8%), followed by motorcycle drivers (135, 25.2%), bicyclists (100, 18.7%), and motor vehicle drivers (50, 9.3%). The average ages of patients were 37.86, 34.53, 37.85, and 49.14 (bicyclists, motorcyclists, motor vehicle occupants, and pedestrians, respectively), and the male-to-female ratios were higher for bicyclists and motorcyclists (13.7:1 and 11.9:1, respectively) compared with motor vehicle occupants and pedestrians (2.3:1 and 1.5:1, respectively). Racial demographics and ethnicity varied among the different modes of transportation. For a full summary of patient demographic information, see Table 1.

### 3.2. Injury Patterns

The majority of patients (99.63%) sustained blunt trauma, while only a small fraction (0.37%) experienced penetrating trauma. In the majority of cases, the head had the highest AIS score with a mean of 3.7, and the median ISS was 20 (interquartile range 14). A total of 80.4% of patients had multi-body region injuries, with 431 occurrences, while single-body region injuries accounted for 19.6%, totaling 105 occurrences.

Across the total patient population, the most prevalent injuries were head injuries and fractures. By overall injury category, fractures were the most common with 1125 occurrences, followed by hemorrhage with 695 occurrences, laceration with 311 occurrences, contusion with 242 occurrences, and abrasion with 104 occurrences. More detailed analysis revealed skull fractures had the highest number of occurrences within the dataset (390), followed by subdural and subarachnoid hemorrhages (302 and 252, respectively), fracture of other facial bones (248), and cervical/thoracic/lumbar fracture (122).

More specifically, subdural hemorrhage with loss of consciousness had the highest prevalence, occurring in 169 cases, followed by fractures of the vault of the skull in 156 cases, subarachnoid hemorrhage with loss of consciousness in 141 cases, and fractures of the base of the skull with 110 cases. A total of 72 patients had multiple rib fractures, 69 patients had scalp lacerations, 53 had scalp contusions, and 60 had lung contusions. Overall, injury patterns among the different modes of transportation showed that brain injuries were the most prevalent, followed closely by fractures, then lacerations, concussions/contusions, and other injuries, including hemothorax/pneumothorax and dislocations. Pedestrians were the only ones to experience amputations, with five occurrences (Table 2).

### 3.3. Clinical Outcomes

The analysis of clinical outcomes in patients involved in CIMVs reveals significant variations based on the mode of transportation used. Among the different groups, pedestrians had the highest overall mortality rate of 24%. In comparison, bicyclists had a lower mortality rate of 8.7%, motorcyclists had a rate of 13.1%, and motor vehicle occupants had a rate of 10.9%. This indicates that pedestrians are more vulnerable to such injuries. Lack of protective gear may be one of the reasons for this vulnerability.

Additionally, only 43.8% of pedestrians were discharged home, while 19% required sub-acute or inpatient rehabilitation, reflecting the severity of their injuries. A small percentage, 2.6%, met the criteria for brain death, with slightly higher rates noted among motorcyclists and pedestrians. Discharges to skilled nursing facilities and other acute care settings were also more common for pedestrians, highlighting their increased need for care after hospitalization.

In contrast, bicyclists had the best outcomes overall, evidenced by the highest discharge rate to home at 68.9%. These findings underscore the importance of implementing targeted preventive measures and tailored post-trauma care strategies, especially for pedestrians who face the most significant risk of severe and prolonged injuries following CIMVs.

Overall mortality was 17.4%, with an additional 14 patients (2.6%) meeting brain death criteria. Between the different transportation modalities, pedestrians had the highest mortality rate at 24.0%, followed by motorcyclists (13.1%), motor vehicle occupants (10.9%), and bicyclists (8.7%). Pedestrians were also the least likely to be discharged to home/shelter at 43.8% compared with bicyclists at 68.9%, motorcyclists at 62.8%, and motor vehicle occupants at 54.3%. A complete summary of clinical outcomes is represented in Table 3.

**Figure 1:**
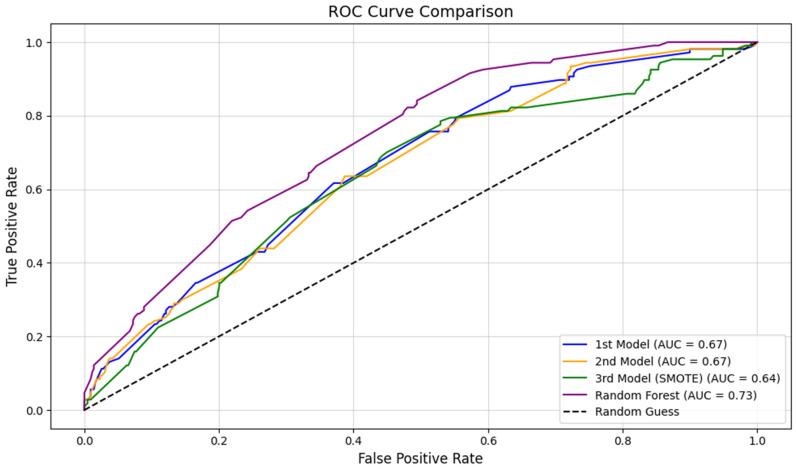
Receiver Operating Characteristic (ROC) curve comparison for four predictive models evaluating discharge outcomes.

This study utilized logistic regression as the primary analysis method because of its suitability for datasets where the dependent variable (Discharge Outcome) and independent variables (Severe TBI-related injuries) are binary-coded (0 for negative, 1 for positive). Logistic regression effectively models the probability of a binary outcome, making it ideal for examining the relationship between severe TBI-related injuries and discharge outcomes. The independent variables were not mutually exclusive, with possible overlapping injuries in trauma patients, as abstracted from the electronic medical record. This characteristic reinforces the appropriateness of logistic regression, as the method accommodates overlapping predictor variables without requiring strict independence. Our ROC curve with different models is illustrated in Figure 1 of this paper.

The ROC curve illustrates the performance of four models: the first logistic regression model, the second model with class weighting, the third model using SMOTE to address the class imbalance, and a Random Forest model. The AUC values highlight key differences in their ability to discriminate between positive and negative discharge outcomes. The Random Forest model achieved the highest AUC (0.73), outperforming the logistic regression models. Random Forest was included to evaluate whether a non-linear algorithm could better capture complex relationships between overlapping independent variables and the binary outcome. Its performance suggests that Random Forest’s ability to handle interactions and non-linearities between predictors provided an advantage over logistic regression in this dataset.

Despite Random Forest’s better AUC, logistic regression remains a valuable method for its interpretability. The ability to estimate odds ratios provides actionable insights into the contribution of each independent variable (e.g., specific severe TBI-related injuries) to discharge outcomes, which is crucial for clinical decision-making. However, the slightly lower AUC values for logistic regression models (0.67 for the first and second models and 0.64 for the SMOTE model) indicate room for improvement, especially in capturing true positives for the minority class.

The SMOTE-optimized logistic regression model demonstrated improved recall for Class 1 (positive outcomes), addressing the challenge of class imbalance inherent in this dataset. However, its reduced AUC highlights the trade-off between improving recall and maintaining overall discriminative ability (Table 4).

The analysis highlights key predictors of discharge outcomes based on logistic regression models (1st, 2nd, and SMOTE-optimized) and a Random Forest model. Subarachnoid hemorrhage (SAH) consistently emerged as a significant predictor in all logistic regression models, with increasing coefficients indicating a progressively stronger positive association with the likelihood of discharge, supported by highly significant *p*-values. The high feature importance of SAH in the Random Forest model corroborates its predictive relevance. Similarly, Epidural Hematoma (EDH) demonstrated a strong negative association with discharge outcomes across all logistic regression models, as evidenced by its consistently large negative coefficients and highly significant *p*-values, while ranking highly in Random Forest feature importance. Skull fractures (base, occiput, and vault) displayed consistent significance in the second and third logistic regression models, with coefficients indicating a positive association with discharge outcomes and moderate feature importance in the Random Forest model.

Variables such as cerebral edema were significant in the first and second models, where coefficients indicated a positive relationship with discharge, but this significance was lost in the SMOTE-optimized model. Cerebellum hemorrhage and diffuse traumatic brain injury had marginal or inconsistent significance across logistic regression models, with feature importance in the Random Forest model suggesting potential non-linear contributions. Cerebral hemorrhage was not significant in logistic regression but had moderate importance in the Random Forest model, indicating its relevance in capturing complex interactions. These findings underscore the utility of coefficients in logistic regression for assessing the magnitude and direction of associations. At the same time, Random Forest’s feature is important as it provides complementary insights into variable contributions, particularly for non-linear and interaction effects. Combining these linear and non-linear approaches could enhance predictive accuracy while maintaining clinical interpretability.

## 4. Discussion

This retrospective analysis of patients involved in CIMVs from 1 January 2016 and 31 December 2023 provides critical insights into the injury patterns and clinical outcomes associated with various transportation modalities. By examining 536 patients, this study offers a detailed understanding of the distribution of head injuries, fractures, and associated trauma across four primary transportation groups: pedestrians, bicyclists, motorcyclists, and motor vehicle occupants.

Almost half of CIMV trauma patients were pedestrians, one-quarter were motorcyclists, 19.4% were bicyclists, and 8.6% were motor vehicle occupants. This is not similar to the previous literature, which found pedestrians were 10.5–28% of CIMV patients, motorcyclists 21–60.8%, bicyclists 0.7%, and motor vehicle occupants 28.7–51%, depending on the study [13,14,15]. This variation shows that transportation varies widely by location, and as such, preventative measures should be tailored to the individual needs of that location. In the case of this study, measures that protect pedestrians would likely be most beneficial, especially as pedestrians had the highest mortality rate.

The majority of patients in the study were male (75%), which is consistent with prior studies [14,16]. This was even more pronounced in the bicyclist and motorcyclist groups, which were 93.2% and 92.2% male, respectively, also consistent with prior studies [17,18]. Of note, Liasidis et al. found that 81.4% of motorcycle passengers were female [19], compared to drivers, who were majority male. Regardless, this suggests that males have a much higher risk than females of being involved in CIMVs causing traumatic injuries, especially bicyclists and motorcyclists. Yan et al. suggest a possible reason for this difference, finding that male bicyclists were more likely to be involved in crashes caused by a failure to obey traffic signals [20,21].

Almost all patients experienced injuries from blunt trauma, and most had multi-body region injuries. The median ISS was found to be 20. Brain injuries were found to be the most common injury pattern across all modalities (subdural hemorrhage > subarachnoid hemorrhage > epidural hemorrhage), followed by fractures, which varied in prevalence depending on the mode of transportation. This overall trend is similar to a previous study, which found that head and external injuries were present in over 60% of patients who experienced motor vehicle trauma [13]. However, when looked at individually, our findings for each mode of transportation are not consistent with prior studies. Generally, brain injuries and facial/skull fractures were much more prevalent in this study compared with prior findings of extremity injuries.

For bicyclists, the most common injury was facial/skull fractures (61.2%), followed by SDH (58.2%), SAH (43.7%), and EDH/another intracranial injury (both 14.6%). In contrast, Kale et al. found long bone fractures to be the most prevalent at 56% [22]; in this study, they only accounted for 7.8% of injuries.

Similarly, injury prevalence for motorcyclists was facial/skull fracture (68.2%), SDH (50.4%), SAH (45.0%), and EDH (14.7%). The prevalence of long bone fractures was slightly increased (also 14.7%) compared to bicyclists. Takalkar et al. specifically studied head injury patterns in motorcyclists and found similar findings, with SDH and SAH being the most prevalent [18]. Other studies varied in their findings; while one found most injuries were sustained to the head/skull [17], others found extremity fractures [19] and contusions [15] to be the most common injuries. One study showed that head injuries and facial fractures were least prevalent in motorcyclists compared with other transportation modalities [13]. In contrast, this study found that motorcyclists had the highest prevalence of facial/skull fractures, and while SDH was lowest in motorcyclists, other brain injuries like SAH and EDH were increased compared with other modalities.

Motor vehicle occupants were the only mode of transportation to have SDH as the most common injury (58.7%), followed by SAH tied with facial/skull fracture (43.5%), and torso/pelvis fracture (21.7%), which had the highest prevalence compared with other modes of transportation. Likewise, Parkinson et al. showed that motor vehicle occupants were more likely to have intra-abdominal injuries compared with pedestrians [23,24]. These injuries are consistent with those commonly caused by seatbelts, which can explain their increased frequency in this population.

Pedestrians again had similar injury patterns: facial/skull fracture (57.8%), followed by SDH (57.0%), SAH (50.0%), long bone fracture (22.9%), and torso/pelvis fracture (13.1%). Again, this is inconsistent with the previous literature, which found lower extremity (specifically tibia-fibula and femur), pelvic, and vertebral fractures to be the most prevalent [22,23,24]. There were similarities, though, when comparing pedestrian injury to the other transportation modalities; pedestrians were more likely to have long bone fractures, including lower limb (tibia-fibula), clavicle, and radio-ulnar fractures [23].

Mortality overall was 17.4%, with an additional 2.6% meeting brain death criteria. This is increased compared to other studies that had mortality rates of around 3–5% [16,17,23]. Pedestrians had the highest mortality rate at 24.0% and were the most likely to be discharged to sub-acute/inpatient rehabilitation (19.0%) and the least likely to be discharged home (43.8%). This suggests that pedestrians experience more severe and life-altering injuries compared to other transportation groups, necessitating extended care in sub-acute rehabilitation or skilled nursing facilities. Previous studies also found that pedestrian mortality was increased compared to motorcyclists and motor vehicle occupants, with rates ranging from 20–38.4% [25,26]. This presents a unique problem to try to solve. While other transport modalities utilize features to minimize or decrease injury (airbags and seatbelts in motor vehicles, helmets, and protective clothing for bicyclists and motorcyclists), pedestrians have limited protection options against injury in CIMVs [27,28,29]. Analysis of the environment is needed to determine specific risk factors for this pedestrian population [30,31,32]. This may include specific location (intersection/street), time of day, and vehicle type/speed at impact, among other factors.

Motorcyclist and bicyclist mortality were 13.1% and 8.7%, respectively, which is increased compared to prior studies that had mortality rates of 0–4.4% [19,26]. This suggests there are specific risk factors in this population that are leading to increased mortality [33,34,35]. Further study is needed to determine if this is related to the lack of use of protective gear such as helmets or another cause to determine the best way to decrease mortality.

Interestingly, motor vehicle occupants had a relatively low mortality rate (10.9%) and made up the smallest proportion of occurrences (8.6%). Other studies reported increased occurrences [36,37] and had varying mortality rates [38,39]. This finding highlights the effectiveness of safety features such as seatbelts, airbags, and advanced vehicle crumple zones in reducing mortality among vehicle occupants. These findings align with those reported in other studies [40,41,42]. However, the comparatively low discharge-to-home rate (54.3%) indicates that many motor vehicle occupants still experience significant injuries that require prolonged rehabilitation. One explanation for this finding is that the majority of motor vehicle occupants involved in crashes receive minor injuries that do not meet trauma activation requirements, which is why the number of occurrences is low. Those that do meet trauma activation requirements, however, tend to have more severe injuries, resulting in the need for more long-term care.

Though New York City has had success in reducing the number of traffic injuries [6], this study shows that further work needs to be implemented. More so, this study highlighted the different injury patterns and mortality rates between transportation modalities. Prior research has shown that effective strategies for reducing pedestrian traffic injuries rely largely on reducing interactions between pedestrians and motorists. Community-based programs, such as those that provide education to both adults and children, have seen up to a 54% reduction in injuries [43]. Another study showed that reducing traffic volume by 30% also reduced pedestrian injury by 35% [44].

The high incidence of traumatic brain injuries in this study highlights the need for head protection, such as helmets. Unfortunately, helmet usage in New York City is low despite extensive regulations requiring their use. One study found that only 11% of riders who were required to wear a helmet used one [45], and multiple studies have shown that helmet usage is particularly low among bike-share users [46,47]. This shows that regulations and laws are not sufficient to encourage helmet use and further steps are needed, such as education and increasing access to helmets. One study suggested that most modern traffic accidents are due to the limited ability of the human brain to process and react to information quickly enough, and therefore, further prevention strategies should be focused on improving technology to decrease reliance on human decision-making [48].

## 5. Strength

This study is notable compared to previous research on CIMV-related trauma because it provides a detailed analysis of specific injury patterns across various transportation modes, including pedestrians, bicyclists, motorcyclists, and vehicle occupants, within a densely populated urban area. Previous studies provide a general overview of CIMV injuries but do not examine the specific patterns linked to each mode of transportation or only look at one or two modes of transportation in isolation. This research offers a more detailed understanding of how different transportation modes influence injury type and severity. Additionally, studying all transportation modes together allows for comparison between them, highlighting details that might otherwise be missed. For example, the relatively low incidence of motor vehicle occupants compared with the high incidence of pedestrians highlights the need for pedestrian-focused intervention.

The research spans over seven years, resulting in a robust dataset that enhances the reliability of the observed trends and injury associations. Additionally, this study uses logistic regression and includes four models: the first logistic regression model, the second model with class weighting, the third model using SMOTE to address the class imbalance, and a Random Forest model. The AUC values indicate significant differences in the models’ abilities to distinguish between positive and negative discharge outcomes. The Random Forest model achieved the highest AUC of 0.73, surpassing the logistic regression models. The first and second models were significant for injury patterns like cerebral edema. Whereas, Subarachnoid Hemorrhage (SAH) was a significant predictor in all logistic regression models, indicating a progressively stronger positive association with the likelihood of discharge, supported by highly significant *p*-values. Our study is unique not only in terms of studying a broad spectrum of mechanisms of injury but also in terms of using different models. Future studies should focus on improving the prediction of discharge outcomes in patients with severe traumatic brain injury (TBI) by considering factors such as demographics, underlying health conditions, and treatment approaches. Researchers should adopt methods that effectively manage the complexities of injury relationships and address issues like imbalanced data to better identify high-risk patients. Balancing accurate predictions with meaningful data patterns is essential. These efforts will enhance the reliability of future research and provide valuable insights for clinical decision-making in severe TBI cases.

This offers a clearer perspective on the relationships involved. Such methodological advancements facilitate a more precise and actionable understanding of CIMV injuries, which is essential for developing targeted preventive measures and improving trauma care protocols specific to each transportation mode.

## 6. Limitations

This study is limited by its retrospective nature and reliance on data from a single trauma center. Data were collected from the NTRACS Trauma Registry, which may not always provide complete or detailed information. This may limit the generalizability of the findings to other settings with different demographic and socioeconomic characteristics. As such, the findings may not be generalizable to other regions with different transportation patterns or trauma care systems. The study also relies on the accuracy of ICD codes and EHR documentation, which may introduce coding errors or misclassification of injury types. Additionally, this study only included patients who met trauma activation criteria after arriving at the hospital, excluding both patients with more minor injuries and those who died at the scene. Future studies should consider prospective designs and multi-center collaborations to provide a more comprehensive understanding of injury patterns across different settings.

## 7. Conclusions

In conclusion, this study provides valuable insights into the patterns of injury and clinical outcomes among trauma patients involved in CIMVs at a level I trauma center in New York City. Our findings highlight significant differences in injury severity, brain injuries, and fracture patterns across various transportation modalities, with pedestrians, motorcyclists, and bicyclists experiencing more severe and life-threatening injuries compared with motor vehicle occupants. Pedestrians, in particular, exhibited the highest mortality rates and were more likely to require long-term rehabilitation.

Overall, the discrepancy between these findings and previous literature suggests that this is a unique population and that more tailored research needs to be conducted to identify the specific risk factors within each mode of transportation. More specifically, the higher incidence of pedestrian injuries in this study demonstrates the need for targeted reduction strategies. The prevalence of head injuries in this study, coupled with their lack of use in this population, is another example. Future research should focus on identifying specific environmental risk factors and developing targeted interventions to minimize the severity of injury and improve clinical outcomes associated with CIMV-related trauma. These risk factors may be associated with, but are not limited to, urban planning, traffic control, and adherence to safety practices. By addressing these factors, we can move toward safer urban transportation systems and more effective trauma care.

## Figures and Tables

**Table 1:** It shows a correlation between patient demographics and the mode of transportation associated with their injury.

	Count (%) *				
Demographic	Total *n* = 536	Bicyclist *n* = 100	Motorcyclist *n* = 135	Motor Vehicle *n* = 50	Pedestrian *n* = 251
*Gender*					
Female	134 (25.0%)	7 (7.0%)	10 (7.4%)	15 (30.0%)	102 (40.6%)
Male	402 (75.0%)	93 (93.0%)	125 (92.6%)	35 (70.0%)	149 (59.4%)
*Race*					
Asian	66 (12.3%)	11 (11.0%)	7 (5.2%)	3 (6.0%)	45 (17.9%)
Black	23 (4.3%)	4 (4.0%)	6 (4.4%)	6 (12.0%)	7 (2.8%)
Native Hawaiian or Other Pacific Islander	3 (0.6%)	1 (1.0%)	0 (0.0%)	0 (0.0%)	2 (0.8%)
Other	329 (61.4%)	62 (62.0%)	98 (72.6%)	29 (58.0%)	140 (55.8%)
Unknown	33 (6.2%)	7 (7.0%)	7 (5.2%)	7 (14.0%)	12 (4.8%)
White	82 (15.3%)	15 (15.0%)	17 (12.6%)	5 (10.0%)	45 (17.9%)
*Ethnicity*					
Hispanic Origin	230 (42.9%)	51 (51.0%)	78 (57.8%)	14 (28.0%)	87 (34.7%)
Non-Hispanic Origin	252 (47.0%)	39 (39.0%)	50 (37.0%)	25 (50.0%)	138 (55.0%)
Unknown	54 (10.1%)	10 (10.0%)	7 (5.2%)	11 (22.0%)	26 (10.4%)
*Age in Years: Mean (SD)*	42.30 (19.79)	37.86 (15.78)	34.53 (13.21)	37.85 (20.14)	49.14 (21.80)

* Except where otherwise noted. SD: Standard Deviation.

**Table 2:** It illustrates various patterns of brain injuries and fractures related to the mode of transportation in patients with trauma from collisions involving motor vehicles (CIMVs).

	Count (%)				
Injury *	Total *n* = 536	Bicyclist *n* = 103	Motorcyclist *n* = 129	Motor Vehicle *n* = 46	Pedestrian *n* = 258
*Brain Injury Patterns*					
Subdural Hemorrhage (SDH)	299 (55.8%)	60 (58.2%)	65 (50.4%)	27 (58.7%)	147 (57.0%)
Epidural Hemorrhage (EDH)	69 (12.9%)	15 (14.6%)	19 (14.7%)	3 (6.5%)	32 (12.4%)
Subarachnoid Hemorrhage (SAH)	252 (47.0%)	45 (43.7%)	58 (45.0%)	20 (43.5%)	129 (50.0%)
Brain Stem Hemorrhage	9 (1.7%)	1 (0.97%)	4 (3.1%)	1 (2.2%)	3 (1.2%)
Diffuse Traumatic Brain Injury	20 (3.7%)	1 (0.97%)	9 (7.0%)	2 (4.3%)	8 (3.1%)
Other Intracranial Injuries/Hemorrhage of cerebrum, others	63 (11.8%)	15 (14.6%)	18 (14.0%)	6 (13.0%)	24 (9.3%)
*Major Fracture Patterns*					
Skull (Vault, Sphenoid, Parietal, Temporal, and Occipital) Fractures and Facial (Nasal, Mandible, Orbit, Maxilla, and Zygomatic)	320 (59.7%)	63 (61.2%)	88 (68.2%)	20 (43.5%)	149 (57.8%)

* Not mutually exclusive; patients may have >1 injury type.

**Table 3:** This table analyzes clinical outcomes in patients involved in CIMVs based on their mode of transportation.

	Count (%)				
Clinical Outcomes	Total *n* = 536	Bicyclist *n* = 103	Motorcyclist *n* = 129	Motor Vehicle *n* = 46	Pedestrian *n* = 258
Overall Mortality	93 (17.4%)	9 (8.7%)	17 (13.1%)	5 (10.9%)	62 (24.0%)
Met Brain Death Criteria	14 (2.6%)	2 (1.9%)	4 (3.1%)	1 (2.2%)	7 (2.7%)
Discharge to Home/Shelter	290 (54.1%)	71 (68.9%)	81 (62.8%)	25 (54.3%)	113 (43.8%)
Hospice Care	2 (0.37%)	0 (0.0%)	0 (0.0%)	0 (0.0%)	2 (0.78%)
Inpatient Psychiatry Care	1 (0.19%)	0 (0.0%)	0 (0.0%)	0 (0.0%)	1 (0.39%)
Left Against Medical Advice	10 (1.9%)	3 (2.9%)	6 (4.5%)	0 (0.0%)	1 (0.39%)
Other Acute Care Hospital Emergency Departments or Inpatient Facility	27 (5.0%)	4 (3.9%)	1 (0.78%)	7 (15.2%)	15 (5.8%)
Police Custody/Jail/Prison	4 (0.75%)	0 (0.0%)	2 (1.6%)	1 (2.2%)	1 (0.39%)
Skilled Nursing Facility	12 (2.2%)	1 (0.97%)	3 (2.3%)	1 (2.2%)	7 (2.7%)
Sub-Acute Rehabilitation/Inpatient Rehabilitation	83 (15.5%)	13 (12.6%)	15 (11.6%)	6 (13.0%)	49 (19.0%)

**Table 4:** It represents the comparison of coefficients, *p*-values, and feature importance for predictors of discharge outcomes across logistic regression models (1st, 2nd, and SMOTE-optimized) and Random Forest model.

Variable	1st Model			2nd Model			3rd Model—SMOTE			4th Model—Random Forest
	Coefficient	*p*-Value	Standardized Coefficient	Coefficient	*p*-Value	Standardized Coefficient	Coefficient	*p*-Value	Standardized Coefficient	Importance
Constant	−1.671	4.59 × 10^−12^	−1.671	−0.376	0.01504	−0.376	−0.111	0.463646	−0.111	-
SAH	0.567	0.0156730	1.135	0.589	7.70 × 10^5^	1.179	0.831	7.64 × 10^−8^	1.663	0.190460
EDH	−0.971	0.0221173	−2.896	−0.946	0.000156	−2.822	−2.099	3.90 × 10^−9^	−6.262	0.158050
Cerebral edema	0.870	0.0375097	3.844	1.158	8.70 × 10^−5^	5.115	0.427	0.203848	1.886	0.095337
SDH	−0.444	0.0580313	−0.892	−0.386	0.011946	−0.775	−0.598	0.000146	−1.202	0.173765
Cerebellum hemorrhage	2.245	0.0723372	26.066	2.040	0.059744	23.681	1.545	0.214954	17.937	0.046707
Skull, base, occipital and vault fractures	0.413	0.0750466	0.825	0.422	0.004783	0.843	0.367	0.014730	0.733	0.125681
Cerebral contusion and laceration	0.452	0.2749924	1.881	0.245	0.399625	1.016	−0.718	0.065901	−2.983	0.058839
Diffuse traumatic brain injury	−0.431	0.5100574	−2.331	−0.139	0.722206	−0.753	−1.807	0.005424	−9.764	0.073620
Cerebral hemorrhage	0.032	0.9447329	0.124	0.265	0.360797	1.043	−0.566	0.104447	−2.230	0.077541

## Data Availability

The data were requested from the Elmhurst Trauma Registry and extracted using electronic medical records after receiving approval from the Institutional Review Board at our facility (Elmhurst Hospital Center).
